# Anti-tumor roles of both strands of the *miR-455* duplex: their targets *SKA1* and *SKA3* are involved in the pathogenesis of renal cell carcinoma

**DOI:** 10.18632/oncotarget.25410

**Published:** 2018-06-01

**Authors:** Yasutaka Yamada, Takayuki Arai, Satoko Kojima, Sho Sugawara, Mayuko Kato, Atsushi Okato, Kazuto Yamazaki, Yukio Naya, Tomohiko Ichikawa, Naohiko Seki

**Affiliations:** ^1^ Department of Functional Genomics, Chiba University Graduate School of Medicine, Chiba, Japan; ^2^ Department of Urology, Chiba University Graduate School of Medicine, Chiba, Japan; ^3^ Department of Urology, Teikyo University Chiba Medical Center, Ichihara, Japan; ^4^ Department of Pathology, Teikyo University Chiba Medical Center, Ichihara, Japan

**Keywords:** microRNA, miR-455, anti-tumor, renal cell carcinoma, SKA

## Abstract

Recent studies revealed that some passenger strands of miRNAs acted as anti-tumor or oncogenic miRNAs in cancer cells. In this study, we focused on *miR-455-5p* (the passenger strand) and *miR-455-3p* (the guide strand) based on microRNA (miRNA) expression signatures of cancer cells. Both *miR-455-5p* and *miR-455-3p* were downregulated in renal cell carcinoma (RCC) tissues and low expression of these miRNAs was significantly associated with poor prognosis. Cancer cell proliferation, migration and invasive abilities were significantly inhibited by ectopic expression of *miR-455-5p* and *miR-455-3p*. To identify their oncogenic targets, we applied a combination of genome-wide gene expression and *in silico* miRNA database analyses. We focused on spindle and kinetochore-associated proteins, *SKA1* and *SKA3* and demonstrated direct regulation of *SKA1* by *miR-455-5p* and *SKA3* by *miR-455-3p* in RCC cells. Our present data demonstrated overexpression of *SKA3* in RCC clinical specimens. Moreover, the study showed that the *miR-455-3p*/*SKA3* axis contributed to cancer cell aggressiveness. Analytic strategies based on anti-tumor miRNAs, including passenger strands of miRNAs, are effective approaches for the elucidation of the molecular pathogenesis of RCC.

## INTRODUCTION

Renal cell carcinoma (RCC) is the most common kidney-associated neoplasm. Among them, clear cell RCC is the most frequent type, accounting for 70-80% of cases [1]. RCC constitutes 2-3% of human cancers, and the proportion is increasing. Worldwide, more than 350,000 people were diagnosed with RCC and 140,000 people died in 2013 [2]. Treatment for localized RCC is mainly surgical resection, which has a good prognosis, however, the prognosis for metastatic RCC at diagnosis remains poor. Treatments for RCC have grown more sophisticated. For example, since 2000, molecularly targeted therapies have focused on inhibition of angiogenesis, and more recently anti-PD-1 antibodies have been used to activate tumor immunity. However, the therapeutic outcomes fall short, and the 5-year survival of advanced RCC is still around 20% [3, 4]. Thus, discovery of new molecular targets and new treatment strategies for RCC are urgently required.

MicroRNA (miRNA) is a type of small non-coding RNA. It fine tunes expression of RNA transcripts (both protein coding and non-protein coding genes) in a sequence-dependent manner [5]. A single miRNA can control a vast number of RNA transcripts in normal and diseased cells [6]. Therefore, aberrantly expressed miRNAs can break down regulated RNA networks and contribute to cancer cells’ development, metastasis and drug resistance [7].

The traditional description of miRNA function indicates that 1 strand of the miRNA duplex is incorporated into the RNA-induced silencing complex (RISC), becoming the active strand (guide strand), whereas the other strand is degraded and has no function (passenger strand or miRNA^*^) [8]. However, more recent studies of miRNA biogenesis have shown that some miRNA passenger strands are functional in plant and human cells [9]. Analyses of our original miRNA expression signatures by RNA-sequencing revealed that some passenger strands of miRNAs were significantly downregulated in several types of cancers. Based on our signatures, we have found that some passenger strands target oncogenic genes e.g., *miR-144-5p*, *miR-145-3p*, *miR-149-3p*, *miR-150-3p* and *miR-199a/b-3p* [10–16].

In this study, we focused on both *miR-455-5p* (the passenger strand) and *miR-455-3p* (the guide strand) that derived from *miR-455* duplex based on miRNA expression signatures of human cancers [17]. Interestingly, low expression of these miRNAs was significantly associated with poor prognosis of patients with RCC (*miR-455-5p*: *p* = 0.00204 and *miR-455-3p*: *p* = 0.0254) based on cohort data in The Cancer Genome Atlas (TCGA). Here, we investigated the anti-tumor roles of these miRNAs and their respective targeted oncogenic genes in RCC pathogenesis. Our present data showed that both *miR-455-5p* and *miR-455-3p* acted as anti-tumor miRNAs in RCC cells. To identify targeted oncogenes in RCC cells, we studied 27 genes, 15 of which were regulated by *miR-455-5p* and 12 by *miR-455-3p*. We found that they were significantly associated with poor prognosis by TCGA analyses.

The involvement of miRNA passenger strands in cancer pathogenesis is a novel concept in studies of miRNA biogenesis and cancer research. Identification of the function of passenger strands will enhance our understanding of the molecular pathways underlying RCC pathogenesis.

## RESULTS

### Expression levels of *miR-455-5p* and *miR-455-3p* in RCC clinical specimens

**Figure 1:**
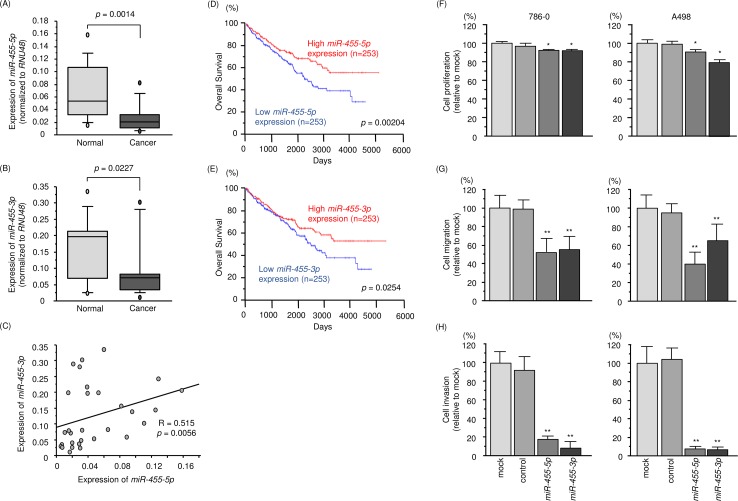
Expression level, clinical significance and anti-tumor function of *miR-455-5p* and *miR-455-3p* in RCC **(A, B)** Expression levels of *miR-455-5p* and *miR-455-3p* in RCC clinical specimens. *RNU48* was used as an internal control. **(C)** Spearman's rank test showed a positive correlation between the expression of *miR-455-5p* and *miR-455-3p*. **(D, E)** Low expression levels of *miR-455-5p* and *miR-455-3p* were associated with low overall survival (*p* = 0.00204 and *p* = 0.0254, respectively). **(F)** Cell proliferation was determined by XTT assays 72 h after transfection with *miR-455-5p* and *miR-455-3p*. **(G)** Cell migration was determined using wound-healing assays. **(H)** Cell invasion activity was determined using Matrigel assays. ^*^, *p* < 0.01. ^**^, *p* < 0.0001.

The public miRNA database (miRbase: release 21) revealed that *miR-455* is located on chromosome 9q32 and the mature sequence of *miR-455-5p* (passenger strand) was 5’ – uaugugccuuuggacuacaucg – 3’ and that of *miR-455-3p* (guide strand) was 5’ - gcaguccaugggcauauacac – 3’. We investigated the expression of *miR-455-5p* and *miR-455-3p* in clinical RCC tissues (paired cancerous and adjacent non-cancerous tissues). Expression levels of *miR-455-5p* and *miR-455-3p* were significantly downregulated in RCC tissues compared with those in noncancerous tissues (*p* = 0.0014; Figure 1A and p = 0.0227; Figure 1B). Furthermore, Spearman's rank test showed a positive correlation between expression levels of *miR-455-5p* and *miR-455-3p* (*p* = 0.0056, R = 0.515; Figure 1C). To investigate the molecular mechanisms of silencing of *miR-455-5p* and *miR-455-3p* in RCC cells, A498 cells were treated with the demethylating agent [5-aza-2’-deoxycytidine (5-aza-dC)]. Expression of *miR-455-5p and miR-455-3p* were not dramatically elevated by 5-aza-dc treatment (data not shown).

A large cohort analysis (n = 506) based on the TCGA database showed that low expression levels of *miR-455-5p and miR-455-3p* were associated with poor survivals in RCC patients (*p* = 0.00204 and *p* = 0.0254; Figure 1D and 1E, respectively).

### Effects of ectopic expression of *miR-455-5p* and *miR-455-3p* on RCC cells

We performed gain-of-function studies by miRNAs transfection into 786-O and A498 cells. XTT assays revealed that cell proliferation was significantly inhibited in *miR-455-5p* and *miR-455-3p* transfectants compared with that in mock or control transfectants (Figure 1F). Cell migration activity was significantly inhibited in *miR-455-5p* and *miR-455-3p* transfectants in comparison with those in mock or control transfectants (Figure 1G). Likewise, Matrigel assays showed that cell invasion activity was significantly inhibited in *miR-455-5p* and *miR-455-3p* transfectants compared to those in mock or control transfectants (Figure 1H). We further investigated synergistic effects of *miR-455-5p* and *miR-455-3p* expression in RCC cells. As a result, synergistic effects were not identified in this study (Supplementary Figures 1).

### Incorporation of *miR-455-5p* into the RISC in RCC cells

We proposed that passenger strand *miR-455-5p* may be incorporated into the RNA-induced silencing complex (RISC) and thereby have a role in regulating gene activities in cancer cells. To investigate that hypothesis, we performed immunoprecipitation with antibodies targeting Argonaute2 (Ago2), which plays an important role in the RISC. After transfection with *miR-455-5p* or *miR-455-3p*, Ago2-bound miRNAs were isolated, and we performed qRT-PCR to determine whether *miR-455-5p* and *miR-455-3p* were bound to Ago2. After transfection with *miR-455-5p* and immunoprecipitation by anti-Ago2 antibodies, *miR-455-5p* levels were significantly higher than those of mock- or miR-control-transfected cells and those of *miR-455-3p*-transfected 786-O cells (Supplementary Figure 2A). Likewise, after *miR-455-3p* transfection, *miR-455-3p* was detected by Ago2 immunoprecipitation (Supplementary Figure 2B).

### Searching for putative targets regulated by *miR-455-5p and miR-455-3p* in RCC cells

**Figure 2:**
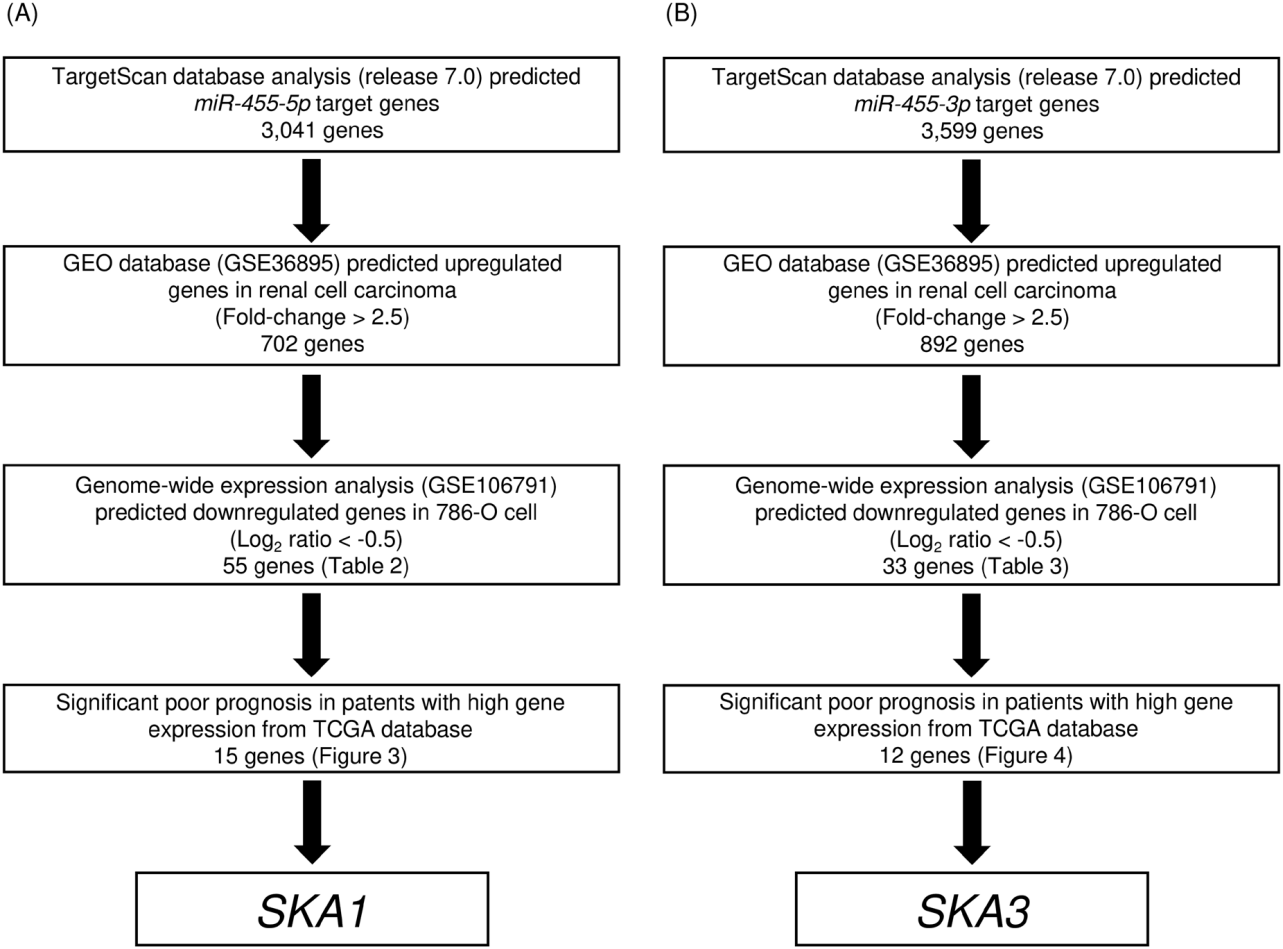
Flow chart illustrating the analytic strategy for identifying *miR-455-5p* and *miR-455-3p* targets in RCC cells A total of 3,041 and 3,599 genes were putative target genes of *miR-455-5p* and *miR-455-3p* in TargetScan database analysis (release 7.0). Of those groups, 15 and 12 genes were identified as putative target genes of *miR-455-5p* and *miR-455-3p* in RCC cells, respectively.

We performed both *in silico* and gene expression analysis to identify genes targeted by *miR-455-5p* and *miR-455-3p* for regulation. The strategy for identification of *miR-455-5p* and *miR-455-3p* target genes is shown in Figure 2A and 2B. First, we identified 3,041 and 3,559 genes that had putative target sites for *miR-455-5p* and *miR-455-3p* in their 3′-UTR according to the TargetScanHuman 7.0 database. Next, we narrowed down those groups to 702 and 892 genes whose expression levels were upregulated (Fold-change > 2.5) in RCC cells using a GEO database (accession number: GSE36895). Next, we identified 55 and 33 genes that were downregulated after *miR-455-5p* and *miR-455-3p* were transfected into 786-O cell (Log_2_ ratio < −0.5; Tables 1 and 2).

**Table 1:** Putative target genes regulated by *miR-455-5p* in RCC cells

Gene Symbol	Gene Name	Conserved sites count	Poorly conserved sites count	GEO expression data Fold-Change (Tumor/Normal)	786-O *miR-455-5p* transfection (Log_2_ ratio)	Cytoband	TCGA data OS (*p* value)
*BIRC5*	baculoviral IAP repeat containing 5	0	1	2.728	-0.888	hs|18q11.2	2.93E-09
*SKA1*	spindle and kinetochore associated complex subunit 1	0	1	3.751	-0.836	hs|20p11.23	1.44E-07
*CDCA8*	cell division cycle associated 8	0	1	3.071	-0.547	hs|17q23.2	2.93E-06
*CENPF*	centromere protein F, 350/400kDa	0	1	2.699	-0.905	hs|9p13.3	7.01E-05
*DEPDC1*	DEP domain containing 1	0	2	2.606	-1.076	hs|9q34.11	0.000111
*ELOVL2*	ELOVL fatty acid elongase 2	0	1	3.11	-0.72	hs|12p13.1	0.000146
*TNFSF4*	tumor necrosis factor (ligand) superfamily, member 4	0	1	2.65	-0.948	hs|17q25.3	0.000257
*CD72*	CD72 molecule	0	1	3.643	-1.117	hs|17q21.2	0.000668
*KIAA0101*	KIAA0101	0	2	3.358	-0.629	hs|16p13.3	0.00258
*PLEKHG4*	pleckstrin homology domain containing, family G (with RhoGef domain) member 4	0	1	2.743	-0.716	hs|12q24.23	0.00298
*TNIP3*	TNFAIP3 interacting protein 3	0	1	4.313	-1.142	hs|11q12.1	0.00339
*FOXL1*	forkhead box L1	0	1	2.987	-2.048	hs|19q13.41	0.0129
*SLC7A11*	solute carrier family 7 (anionic amino acid transporter light chain, xc- system), member 11	0	2	2.677	-1.518	hs|12q23.2	0.0234
*SIRPA*	signal-regulatory protein alpha	0	1	2.737	-1.484	hs|17p11.2	0.0349
*VCAN*	versican	1	1	5.753	-0.865	hs|22q13.31	0.0467
*IL21R*	interleukin 21 receptor	0	1	3.377	-1.068	hs|2q11.2	n.s.
*HLA-DPB1*	major histocompatibility complex, class II, DP beta 1	0	1	2.781	-0.81	hs|5q31.1	n.s.
*LAYN*	layilin	1	1	2.575	-1.356	hs|17p11.2	n.s.
*IKZF1*	IKAROS family zinc finger 1 (Ikaros)	0	1	2.548	-0.519	hs|6p21.1	n.s.
*SLC38A1*	solute carrier family 38, member 1	0	1	3.365	-1.049	hs|12q13.11	n.s.
*BRIP1*	BRCA1 interacting protein C-terminal helicase 1	0	1	2.71	-0.519	hs|22q11.21	n.s.
*GJC1*	gap junction protein, gamma 1, 45kDa	1	0	5.978	-0.895	hs|19q13.2	n.s.
*IGFBP3*	insulin-like growth factor binding protein 3	0	1	11.356	-2.607	hs|12q13.11	n.s.
*LOX*	lysyl oxidase	0	2	9.982	-1.551	hs|15q26.3	n.s.
*EHD2*	EH-domain containing 2	0	1	9.206	-2.008	hs|22q13.1	n.s.
*DIRAS2*	DIRAS family, GTP-binding RAS-like 2	0	1	6.202	-1.342	hs|11q25	n.s.
*LRRC25*	leucine rich repeat containing 25	0	1	6.156	-0.685	hs|11q23.3	n.s.
*RASSF2*	Ras association (RalGDS/AF-6) domain family member 2	0	1	6.146	-1.364	hs|Xq28	n.s.
*GAS2L3*	growth arrest-specific 2 like 3	0	1	5.641	-0.73	hs|17q12	n.s.
*EGFR*	epidermal growth factor receptor	0	1	4.5	-1.689	hs|19q13.2	n.s.
*KRBA1*	KRAB-A domain containing 1	0	2	4.421	-1.144	hs|2p23.3	n.s.
*SLC1A3*	solute carrier family 1 (glial high affinity glutamate transporter), member 3	0	1	4.302	-0.645	hs|22q13.33	n.s.
*TLR3*	toll-like receptor 3	0	1	4.139	-2.606	hs|5q14.3	n.s.
*MS4A7*	membrane-spanning 4-domains, subfamily A, member 7	0	1	4.088	-0.619	hs|19p13.11	n.s.
*PTGS1*	prostaglandin-endoperoxide synthase 1 (prostaglandin G/H synthase and cyclooxygenase)	0	1	4.043	-2.257	hs|12q23.3	n.s.
*FAM111B*	family with sequence similarity 111, member B	0	1	3.986	-0.996	hs|17q21.32	n.s.
*APOLD1*	apolipoprotein L domain containing 1	0	1	3.953	-1.385	hs|6p21.1	n.s.
*CDHR1*	cadherin-related family member 1	0	1	3.727	-0.574	hs|7p12.2	n.s.
*PHKA2*	phosphorylase kinase, alpha 2 (liver)	0	1	3.661	-0.955	hs|15q15.1	n.s.
*LRRK1*	leucine-rich repeat kinase 1	0	1	3.558	-0.654	hs|7q36.3	n.s.
*PGBD5*	piggyBac transposable element derived 5	0	1	3.516	-1.466	hs|6p24.2	n.s.
*LCK*	LCK proto-oncogene, Src family tyrosine kinase	0	1	3.269	-1.235	hs|5p13.2	n.s.
*HS3ST2*	heparan sulfate (glucosamine) 3-O-sulfotransferase 2	0	1	2.985	-1.116	hs|1q21.3	n.s.
*NEURL1B*	neuralized E3 ubiquitin protein ligase 1B	0	1	2.906	-1.008	hs|17q21.32	n.s.
*COL8A1*	collagen, type VIII, alpha 1	0	1	2.88	-1.102	hs|2p22.2	n.s.
*DIAPH2*	diaphanous-related formin 2	0	1	2.872	-0.653	hs|9p13.3	n.s.
*GJA1*	gap junction protein, alpha 1, 43kDa	0	1	2.797	-0.658	hs|12q23.2	n.s.
*LCP1*	lymphocyte cytosolic protein 1 (L-plastin)	0	1	2.773	-2.022	hs|2p16.2	n.s.
*CMPK2*	cytidine monophosphate (UMP-CMP) kinase 2, mitochondrial	0	1	2.747	-0.963	hs|5q31.1	n.s.
*TRPV2*	transient receptor potential cation channel, subfamily V, member 2	0	4	2.701	-0.644	hs|11q12.1	n.s.
*GRAMD4*	GRAM domain containing 4	0	1	2.684	-0.921	hs|3q13.12	n.s.
*SEMA6A*	sema domain, transmembrane domain (TM), and cytoplasmic domain, (semaphorin) 6A	0	2	2.61	-1.354	hs|3p21.31	n.s.
*EDN1*	endothelin 1	0	1	2.598	-0.774	hs|1p34.3	n.s.
*TMEM140*	transmembrane protein 140	0	1	2.558	-0.801	hs|5q35.3	n.s.
*LRP4*	low density lipoprotein receptor-related protein 4	0	1	2.525	-0.96	hs|6q22.31	n.s.

n.s., not significant.

**Table 2:** Putative target genes regulated by *miR-455-3p* in RCC cells

Gene Symbol	Gene Name	Conserved sites count	Poorly conserved sites count	GEO expression data Fold-Change (Tumor/Normal)	786-O *miR-455-3p* transfection (Log_2_ ratio)	Cytoband	TCGA data OS (*p* value)
*TRIM36*	tripartite motif containing 36	0	2	2.822	-1.084	hs|5q22.3	1.66E-06
*FXYD5*	FXYD domain containing ion transport regulator 5	0	1	4.276	-1.377	hs|19q13.12	3.60E-06
*CENPF*	centromere protein F, 350/400kDa	0	1	2.699	-0.652	hs|1q41	7.01E-05
*NCAPG*	non-SMC condensin I complex, subunit G	0	1	2.746	-0.977	hs|4p15.31	7.27E-05
*PARVG*	parvin, gamma	0	1	3.403	-2.019	hs|22q13.31	0.000548
*SKA3*	spindle and kinetochore associated complex subunit 3	0	1	2.597	-0.756	hs|13q12.11	0.000596
*ISG20*	interferon stimulated exonuclease gene 20kDa	0	1	5.168	-0.719	hs|15q26.1	0.0014
*PAQR4*	progestin and adipoQ receptor family member IV	0	1	5.134	-1.213	hs|16p13.3	0.00152
*COL5A1*	collagen, type V, alpha 1	0	1	3.025	-0.547	hs|9q34.3	0.00164
*PLXDC1*	plexin domain containing 1	0	2	3.144	-1.3	hs|17q12	0.00186
*PRR7*	proline rich 7 (synaptic)	0	1	2.503	-0.737	hs|5q35.3	0.00307
*C10orf10*	chromosome 10 open reading frame 10	0	1	3.95	-0.59	hs|10q11.21	0.0456
*PFKP*	phosphofructokinase, platelet	0	1	5.385	-0.511	hs|10p15.2	n.s.
*HK2*	hexokinase 2	0	1	26.667	-0.864	hs|2p12	n.s.
*GRIK3*	glutamate receptor, ionotropic, kainate 3	1	0	6.25	-0.818	hs|1p34.3	n.s.
*HSPG2*	heparan sulfate proteoglycan 2	0	1	5.466	-0.572	hs|1p36.12	n.s.
*ARL11*	ADP-ribosylation factor-like 11	0	1	5.283	-0.813	hs|13q14.2	n.s.
*CXorf36*	chromosome X open reading frame 36	0	2	4.975	-1.576	hs|Xp11.3	n.s.
*FAM57A*	family with sequence similarity 57, member A	0	1	4.89	-0.662	hs|17p13.3	n.s.
*FUT11*	fucosyltransferase 11 (alpha (1,3) fucosyltransferase)	0	1	4.124	-1.519	hs|10q22.2	n.s.
*DDIT4*	DNA-damage-inducible transcript 4	0	1	3.996	-0.561	hs|10q22.1	n.s.
*PPP1R9B*	protein phosphatase 1, regulatory subunit 9B	1	0	3.801	-0.523	hs|17q21.33	n.s.
*TRIM9*	tripartite motif containing 9	0	1	3.763	-0.878	hs|14q22.1	n.s.
*DCLK1*	doublecortin-like kinase 1	0	1	3.633	-1.087	hs|13q13.3	n.s.
*CSF1R*	colony stimulating factor 1 receptor	0	1	3.418	-0.88	hs|5q32	n.s.
*KCNE4*	potassium voltage-gated channel, Isk-related family, member 4	0	1	3.368	-0.867	hs|2q36.1	n.s.
*GPR20*	G protein-coupled receptor 20	0	1	3.196	-1.272	hs|8q24.3	n.s.
*GPR85*	G protein-coupled receptor 85	0	3	2.918	-0.608	hs|7q31.1	n.s.
*ADAMTS2*	ADAM metallopeptidase with thrombospondin type 1 motif, 2	0	1	2.862	-0.679	hs|5q35.3	n.s.
*HLA-DPB1*	major histocompatibility complex, class II, DP beta 1	0	1	2.781	-1.046	hs|6p21.32	n.s.
*BRIP1*	BRCA1 interacting protein C-terminal helicase 1	0	1	2.71	-0.996	hs|17q23.2	n.s.
*PIK3R5*	phosphoinositide-3-kinase, regulatory subunit 5	0	1	2.623	-0.594	hs|17p13.1	n.s.
*IKZF1*	IKAROS family zinc finger 1 (Ikaros)	0	2	2.548	-0.655	hs|7p12.2	n.s.

n.s., not significant.

**Figure 3:**
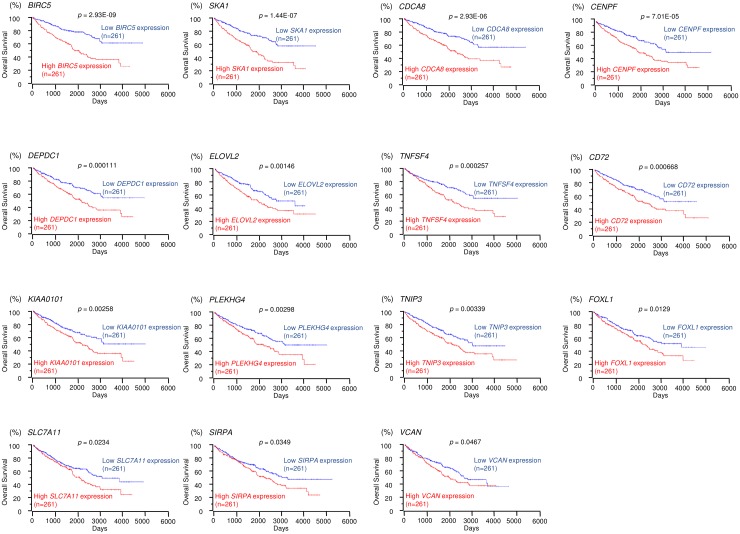
TCGA database analysis of putative targets of *miR-455-5p* in RCC Kaplan-Meier plots of overall survival with log-rank tests for 15 genes with high and low expression from the TCGA database.

**Figure 4:**
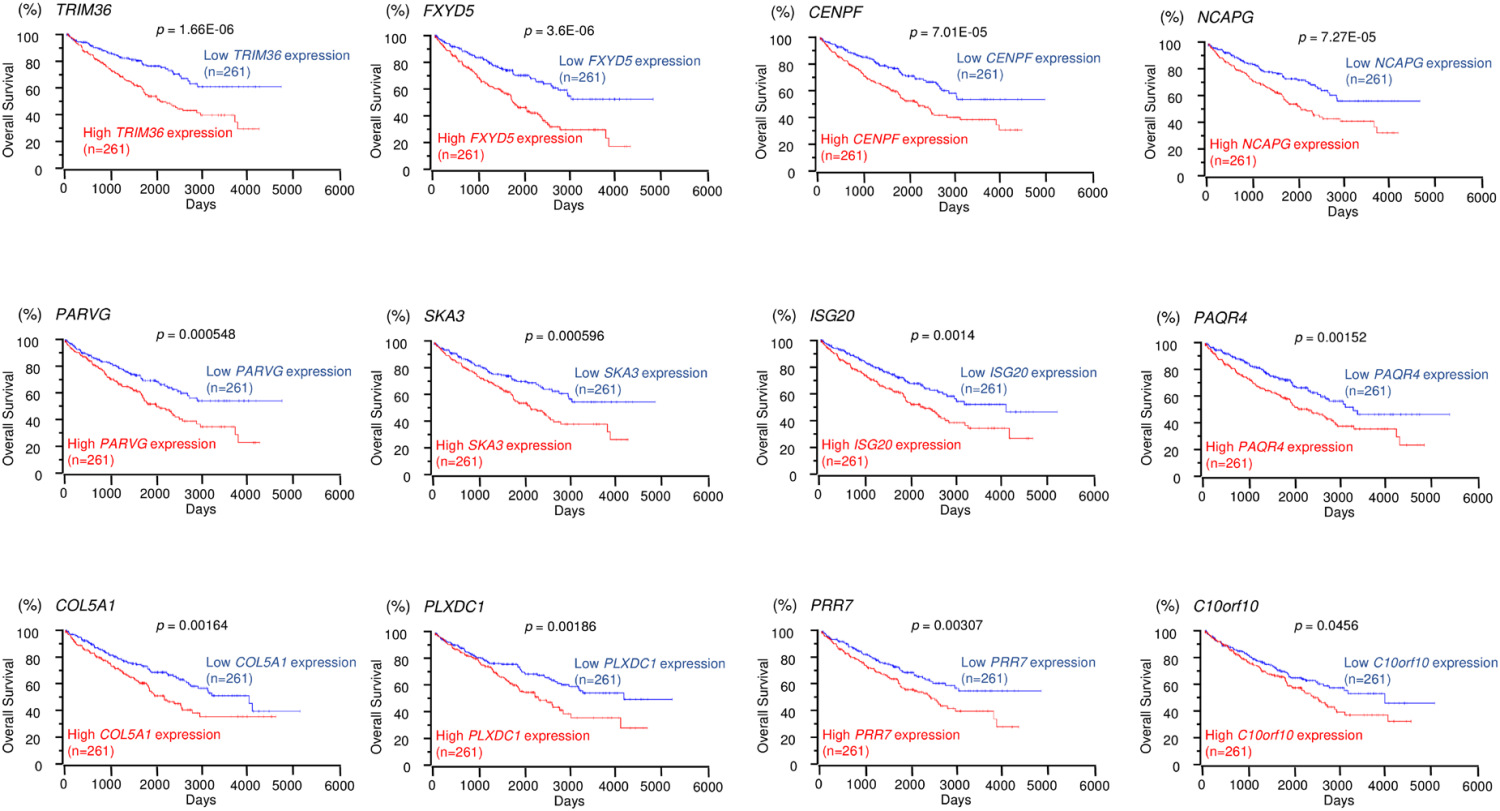
TCGA database analysis of putative targets of *miR-455-3p* in RCC Kaplan-Meier plots of overall survival with log-rank tests for 12 genes with high and low expression from the TCGA database.

We selected 15 and 12 genes whose high expression levels were associated (*p* < 0.05) with low overall survivals of RCC patients according to the OncoLnc database. Kaplan-Meier survival curves showed that high expression levels of 15 and 12 genes were associated with poor prognosis in RCC patients (Figures 3 and 4, respectively). Moreover, we analyzed whether these gene sets (Tables 1 and 2) would be prognostic markers for patients with RCC. Our results showed that expression status of these gene sets were effective as prognostic markers for patients with RCC (Supplementary Figure 3). Patients with high gene signature expressions were significantly associated with short DFS and OS than those with low gene signature expressions (*p* < 0.0001, Supplementary Figures 3A-2, 3A-3, 3B-2 and 3B-3). These findings showed that *miR-455-5p* and *miR-455-3p* regulated molecular networks were deeply involved in RCC pathogenesis and may be therapeutic targets of RCC. Genomics Analysis and Visualization Platform were used for visualization of gene expression heatmaps (http://r2.amc.nl) [18]. The normalized mRNA expression values in the RNA sequencing data were processed and provided as Z-scores.

We focused on spindle and kinetochore-associated complex subunits 1 and 3 (*SKA1* and *SKA3*) because we recently reported that regulation of *SKA1* by anti-tumor *miR-10a-5p* was involved in RCC pathogenesis. Both *SKA1* and *SKA3* were pivotal candidate genes targeted by *miR-455-5p* and *miR-455-3p*, respectively, and we hypothesized that the *SKA* complex was closely involved in RCC pathogenesis and could be regulated by several anti-tumor miRNAs in RCC cells.

### Direct regulation of *SKA1* by *miR-455-5p* and *SKA3* by *miR-455-3p* in RCC cells

**Figure 5:**
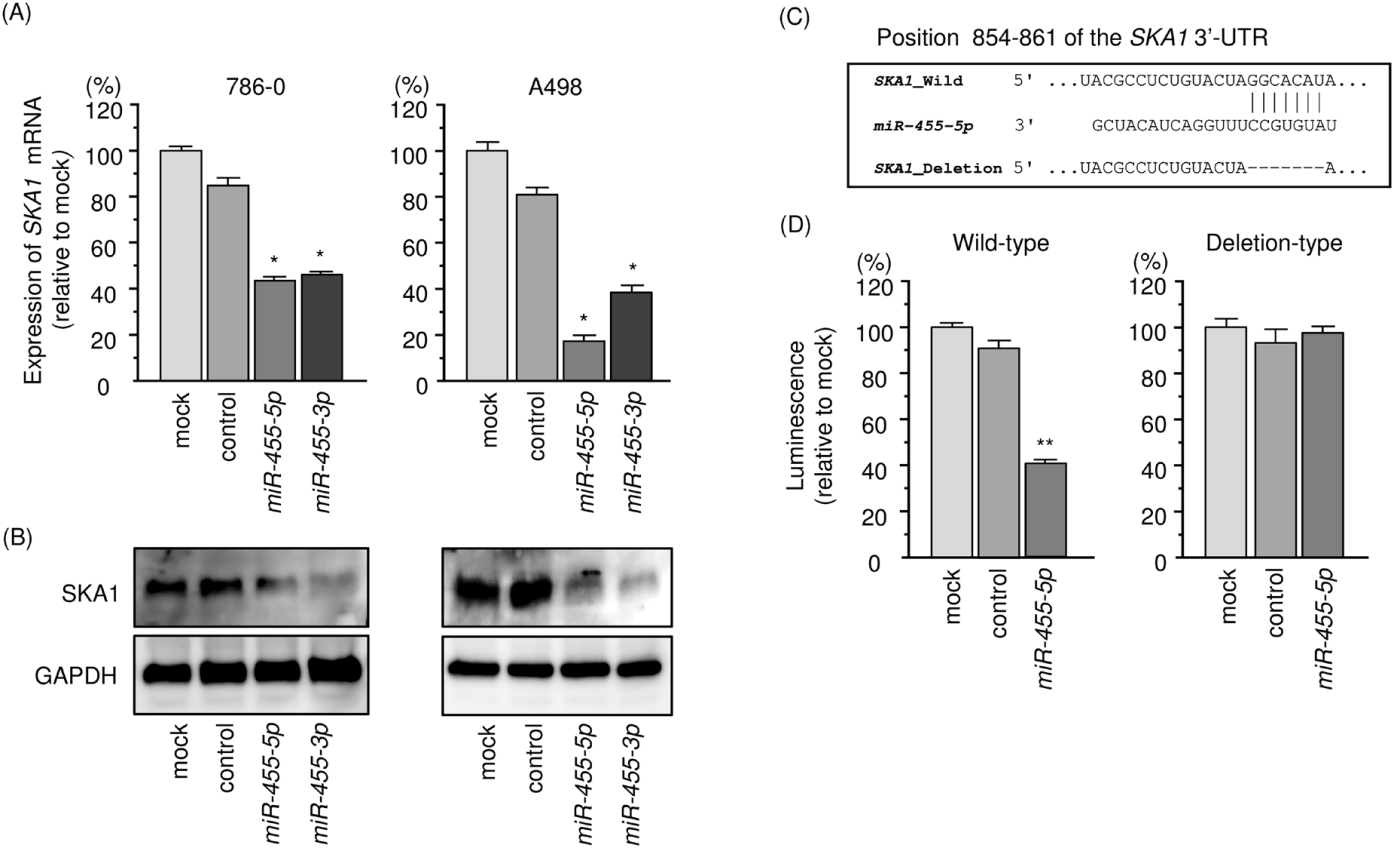
Regulation of *SKA1* expression by *miR-455-5p* in RCC cells **(A)** Expression levels of *SKA1* mRNA 48 h after transfection of 10 nM *miR-455-5p* into cell lines. *GUSB* was used as an internal control. **(B)** Protein expression of SKA1 72 h after transfection of *miR-455-5p*. GAPDH was used as a loading control. **(C)** *miR-455-5p* binding sites in the 3′-UTR of *SKA1* mRNA. **(D)** Dual luciferase reporter assays using vectors encoding putative *miR-455-5p* target sites (positions 854–861) in the *SKA1* 3′-UTR for both wild-type and deleted regions. Normalized data were calculated as the ratio of *Renilla*/firefly luciferase activities. ^*^, *p* < 0.0001. ^**^, *p* < 0.01.

Both mRNA and protein expression levels of *SKA1*/SKA1 were reduced by ectopic expression of *miR-455-5p* in 786-O and A498 cells (Figure 5A and 5B). The TargetScan database shows that the *SKA1* gene has a single target site for *miR-455-5p* in its 3’-UTR region (Figure 5C). No target site for *miR-455-3p* was detected by the TargetScan database. Luminescence intensity was significantly reduced by co-transfection with *miR-455-5p* and the vector carrying the wild-type 3′-UTR of *SKA1* (Figure 5D). Conversely, luminescence intensity was not reduced when the target site of *miR-455-5p* was deleted from the vectors (Figure 5D).

**Figure 6:**
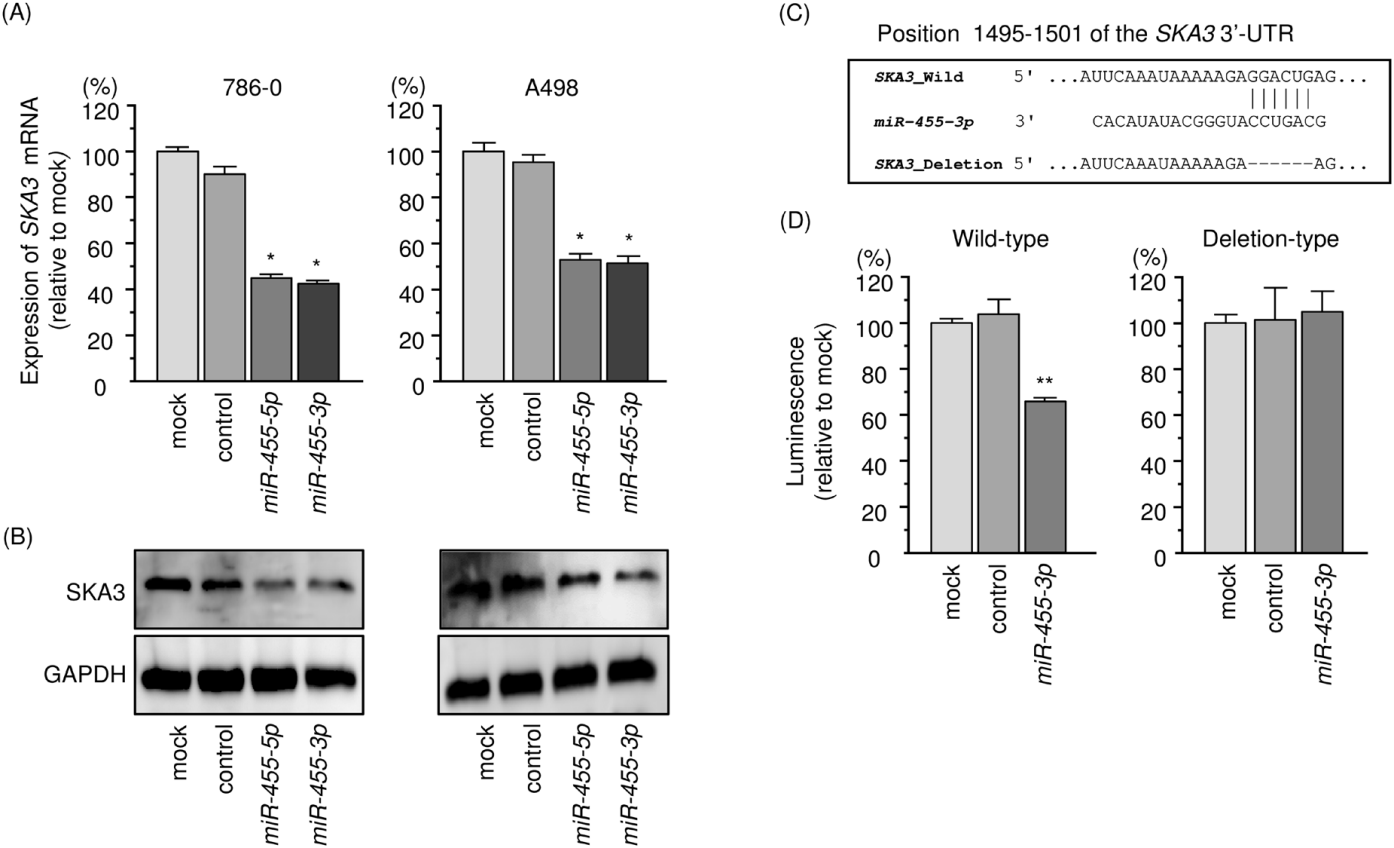
Regulation of *SKA3* expression by *miR-455-3p* in RCC cells **(A)** Expression levels of *SKA3* mRNA 48 h after transfection of 10 nM *miR-455-3p* into cell lines. *GUSB* was used as an internal control. **(B)** Protein expression of SKA3 72 h after transfection with *miR-455-3p*. GAPDH was used as a loading control. **(C)** *miR-455-3p* binding sites in the 3′-UTR of *SKA3* mRNA. **(D)** Dual luciferase reporter assays using vectors encoding putative *miR-455-5p* target sites (positions 1495–1501) in the *SKA3* 3′-UTR for both wild-type and deleted regions. Normalized data were calculated as the ratio of *Renilla*/firefly luciferase activities. ^*^, *p* < 0.0001. ^**^, *p* < 0.01.

In Figure 6A and 6B, the expression levels of *SKA3*/SKA3 were reduced by ectopic expression of *miR-455-3p* in 786-O and A498 cells. A single binding site for *miR-455-3p* is annotated by the TargetScan database in the 3’-UTR region of *SKA3*. However, no binding site for *miR-455-5p* was observed in the 3’-UTR region of *SKA3* (Figure 6C). Luciferase reporter assays showed that luminescence intensity was significantly reduced by co-transfection with *miR-455-3p* and the vector carrying the wild-type 3′-UTR of *SKA3* (Figure 6D).

Unexpectedly in these analyses, expression of *SKA1*/SKA1 was reduced by *miR-455-3*p restoration (Figure 5A and 5B). Likewise, expression of *SKA3*/SKA3 was affected by *miR-455-5*p restoration (Figure 6A and 6B). Based on these data, we proposed that the destruction of SKA1 affects SKA3, and the destruction of SKA3 affects SKA1. To verify this hypothesis, we conducted experiments using si-*SKA1* and si-*SKA3* in RCC cells. Our data showed that the expression levels of *SKA1* were reduced by si-*SKA3* transfection (Supplementary Figure 4A). Similarly, expression of *SKA3* was reduced by si-*SKA1* transfection (Supplementary Figure 4B). From this analysis it became clear that the expression of *SKA1* and *SKA3* mutually influenced each other.

SKA family consists of SKA1, SKA2 and SKA3. In addition, we investigated whether expression of SKA1 or SKA3 affected the expression of SKA2 in RCC cells. Furthermore, expression of *SKA2* was reduced by si-*SKA1* or si-*SKA3* transfection (Supplementary Figure 4C and 4D).

### Effects of silencing *SKA3* in RCC cells

**Figure 7:**
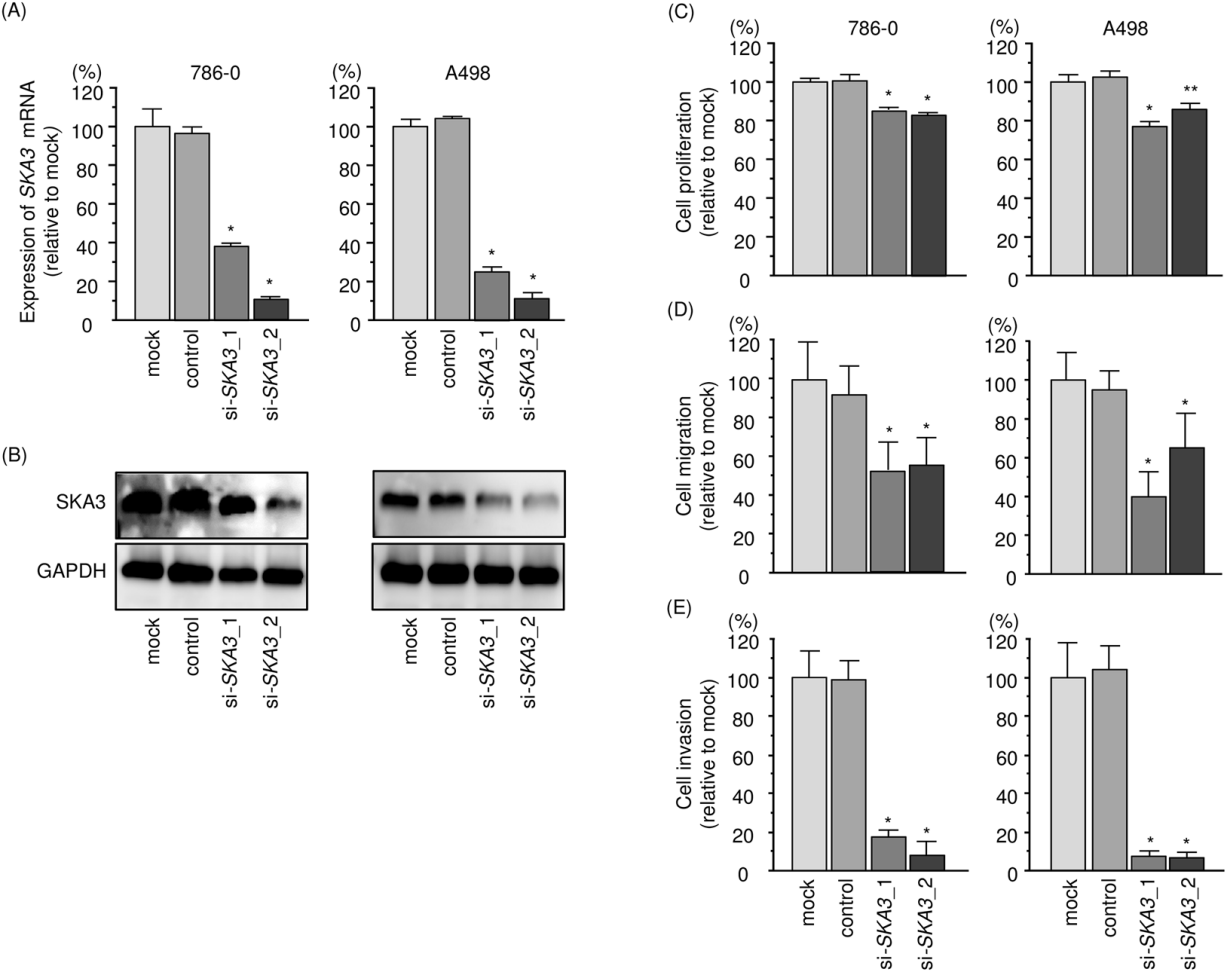
Effects of *SKA3* silencing in RCC cell lines **(A)** *SKA3* mRNA expression 48 h after transfection with 10 nM si-*SKA3* into RCC cell lines. *GUSB* was used as an internal control. **(B)** SKA3 protein expression 72 h after transfection with si-*SKA3*. GAPDH was used as a loading control. **(C)** Cell proliferation was determined with XTT assays 72 h after transfection of 10 nM si-*SKA3*_1 or si-*SKA3*_2. **(D)** Cell migration activity. **(E)** Cell invasion activity. ^*^, *p* < 0.0001. ^**^, *p* < 0.001.

We performed loss-of-function experiments using si-*SKA3* (si-*SKA3*_1 and si-*SKA3*_2) transfection into 786-O and A498 cells to investigate the functional significance of *SKA3* in RCC cells. We demonstrated that the expression levels of *SKA3* mRNA and SKA3 protein were significantly reduced in qRT-PCR and Western blotting analyses (Figure 7A and 7B). Furthermore, functional assays showed that si-*SKA3* transfection significantly inhibited cell proliferation, migration, and invasion in comparison with mock- or si-control-transfected cells (Figure 7C-7E).

### Expression of *SKA3* in RCC clinical tissues

**Figure 8:**
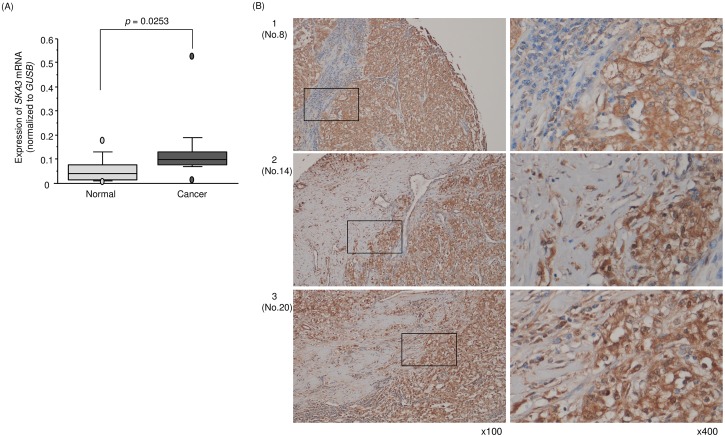
Expression of *SKA3* in clinical specimens of RCC **(A)** Expression levels of *SKA3* in RCC clinical specimens. *GUSB* was used as an internal control. **(B)** Immunostaining showed that SKA3 was strongly expressed in several cancer lesions compared with normal lesions (100× and 400× magnification field).

A total of 15 pairs of RCC tissues plus adjacent noncancerous tissues and RCC cell lines were used to validate the mRNA expression level of *SKA3* by qRT-PCR. Expression of *SKA3* was significantly upregulated in RCC tissues compared with those in adjacent noncancerous tissues (*p* = 0.0253; Figure 8A). Furthermore, we performed immunohistochemistry with an RCC tissue microarray (cat. no. KD806; US Biomax, Rockville, MD, USA). Patient characteristics for samples used in the tissue microarray are described in http://www.biomax.us/tissue-arrays/Kidney/KD806. SKA3 protein was strongly expressed in several cancer lesions whereas it was rarely expressed in normal lesions (Figure 8B).

### Effects of co-transfection of *SKA3* and *miR-455-3p* in RCC cells

**Figure 9:**
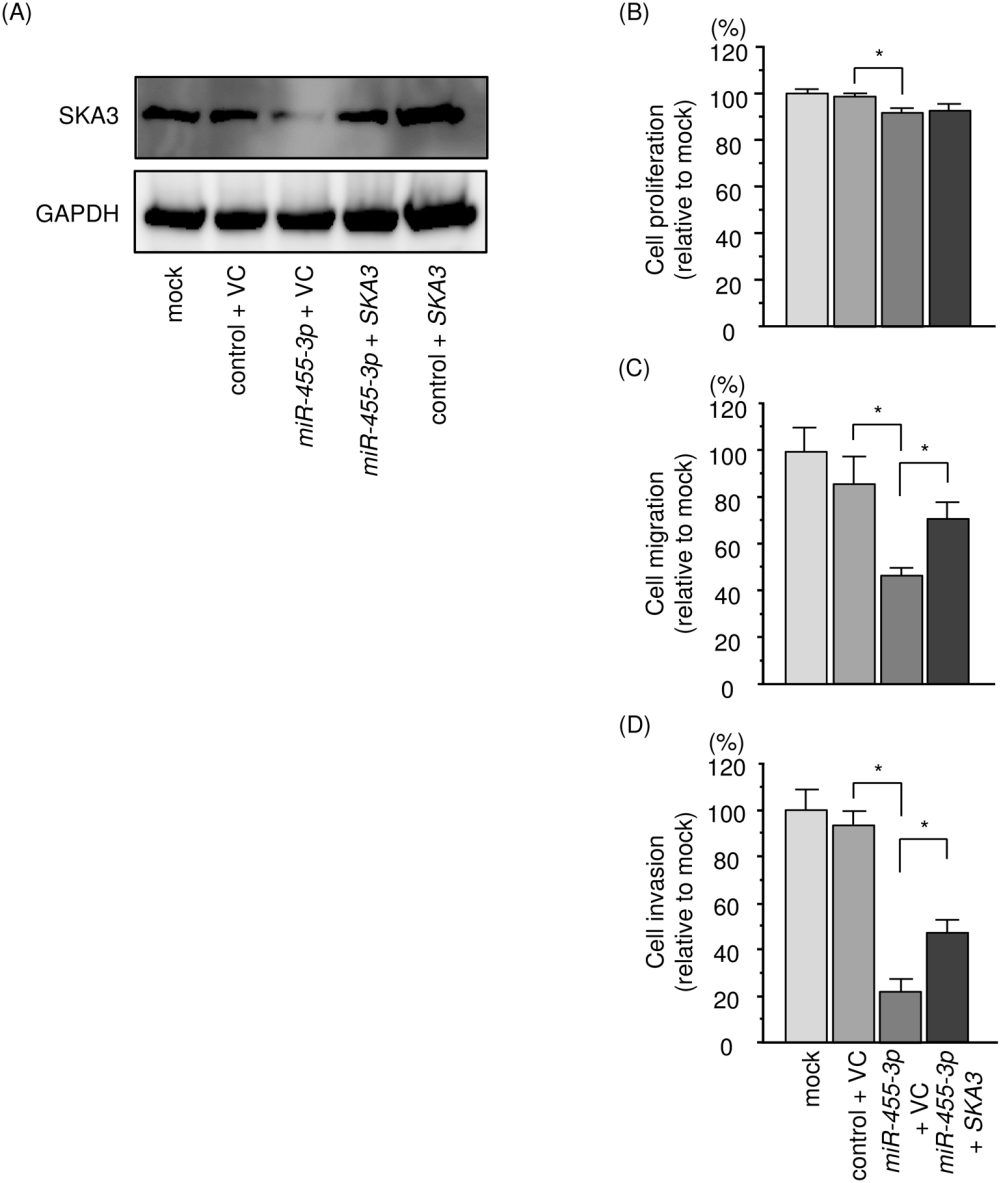
Effects of co-transfection of *SKA3/miR-455-3p* into 786-O cells **(A)** SKA3 protein expression was evaluated by Western blotting analysis of 786-O cells 72 h after reverse transfection with *miR-455-3p* and 48 h after forward transfection with the *SKA3* vector. GAPDH was used as a loading control. **(B)** Cell proliferation was determined using XTT assays 72 h after reverse transfection with *miR-455-3p* and 48 h after forward transfection with the *SKA3* vector. **(C)** Cell migration activity was assessed by wound-healing assays 48 h after reverse transfection with *miR-455-3p* and 24 h after forward transfection with the *SKA3* vector. **(D)** Cell invasion activity was characterized by invasion assays 48 h after reverse transfection with *miR-455-3p* and 24h after forward transfection with *SKA3* vector. ^*^, *p* < 0.0001.

We investigated *SKA3* rescue studies in 786-O cells to elucidate whether the molecular pathway of *SKA3*/*miR-455-3p* was significant for RCC progression. SKA3 protein expression by Western blotting analysis is shown in Figure 9A. Functional assays showed that the proliferation, migration and invasive abilities of RCC cells were significantly recovered by *SKA3* and *miR-455-3p* transfection compared with cells with restored *miR-455-3p* only (Figure 9B-9D). These results suggested that *SKA3* had a pivotal role in RCC progression.

### Clinical significance of the SKA complex in RCC based on TCGA database

**Figure 10:**
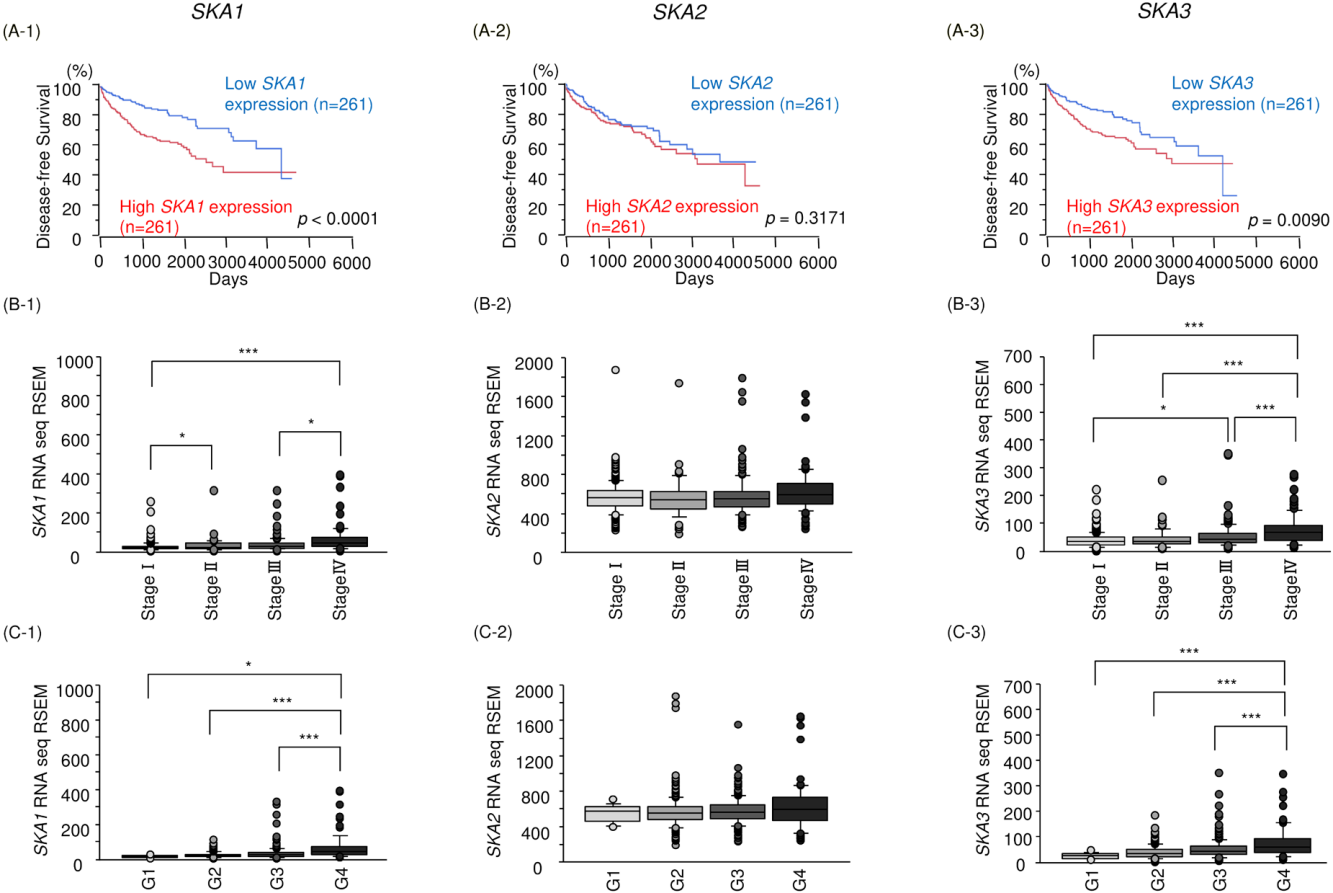
Relationships between expression levels of *SKA1, SKA2* and *SKA3* and disease-free survival, tumor stage and histological grade All patients’ data were obtained from TCGA database. **(A)** Kaplan-Meier survival curves for disease-free survival based on *SKA1, SKA2* and *SKA3* expression in patients with RCC. **(B, C)** Relationships between expression levels of *SKA1, SKA2* and *SKA3* and disease stage and histological grade. ^*^, *p* < 0.01. ^**^, *p* < 0.001. ^***^, *p* < 0.0001.

To validate the clinical significance of SKA complex subunits 1,2 and 3 in RCC pathogenesis, we asked whether the expression levels of *SKA1*, *SKA2* and *SKA3* were associated with the duration of disease-free survival (DFS) in RCC patients. As shown in Figure 10A, high expression levels of *SKA1* and *SKA3* were significantly associated with low DFS in RCC patients. Next, we analyzed the relationships among *SKA1*, *SKA2* and *SKA3* expression and disease stage and histological grade in RCC. *SKA1* and *SKA3* expression levels were significantly increased in the more advance tumor stage and histological grade (Figure 10B and 10C). We further investigated the clinical significance and expression status of *miR-455-5p*/*SKA1* and *miR-455-3p*/*SKA3* in the patients with RCC. Kaplan-Meier analyses showed that low expression of *miR-455-5p*/high expression of *SKA1* group was significantly poor prognosis compared with high expression of *miR-455-5p*/low expression of *SKA1* group by TCGA datasets (Supplementary Figure 5A). In a similar manner, low expression of *miR-455-3p*/high expression of *SKA3* group was predicted poor prognosis compared with high expression of *miR-455-3p*/low expression of *SKA3* group by TCGA datasets (Supplementary Figure 5B).

We performed univariable and multivariable Cox hazard regression analysis to investigate the clinical significance of *SKA1* and *SKA3* expression together with other clinical factors for overall survival in RCC patients. After multivariate analysis, high *SKA1* and *SKA3* expression levels, age, tumor stage and histological grade were independent predictive factors for overall survival (hazard ration (HR) = 1.48, *p* = 0.0134, HR = 1.53, *p* = 0.0073, HR = 2.75, *p* < 0.0001 and HR = 1.67, *p* = 0.004, respectively; Table 3). These results suggested that high expression of *SKA1 and SKA3* is closely associated with cancer progression and the patient's prognosis in RCC.

**Table 3:** Univariable and multivariable Cox hazard regression models for overall survival in RCC patients

Variables	Group	HR	Univariable			Multivariable	
			95% CI	*p* value	HR	95% CI	*p* value
*SKA1* and *SKA3* expression	both high / other	2.04	1.51-2.75	< 0.0001	1.48	1.09-2.02	0.0134
Age	≥60 / <60	1.81	1.33-2.50	0.0001	1.53	1.12-2.12	0.0073
Gender	Male / Female	0.96	0.71-1.32	0.804	-	-	-
Stage	III+IV / I+II	3.74	2.74-5.16	< 0.0001	2.75	1.97-3.90	< 0.0001
Histlogical grade	G3+4 / G1+2	2.61	1.87-3.70	< 0.0001	1.67	1.17-2.42	0.004

## DISCUSSION

Due to recently developed molecularly targeted drugs, RCC treatment outcomes have improved. However, the appearance of drug-resistant cancer cells during the course of treatment is a major obstacle in continued therapy [19]. Discovery of molecular targets for resistant cancer cells has occupied an important position in the development of the latest cancer therapies. To discover novel targets for RCC treatment, we have identified several anti-tumor miRNAs that regulate oncogenic targets in RCC cells, including *miR-26a*, *miR-26b*, *miR-29*a, *miR-29b*, *miR-29c*, *miR-101-3p* and *miR-10a-5p* [17, 20–22]. Our studies revealed that ubiquitin-like with PHD and ring finger domains 1 (*UHRF1*) was directly regulated by *miR-101-3p* and overexpression of *UHRF1* was observed in sunitinib-treated RCC tissues [17]. It was reported that 2 genes (lysyl oxidase-like 2 (*LOXL2*) and procollagen-lysine, 2-oxoglutarate 5-dioxygenase 2 (*PLOD2*)) were direct targets of the *miR-29*-family and *miR-26a*/*miR-26b*, respectively. Overexpression of these genes enhanced cancer cell migration and invasive abilities [20]. More recently, the gene encoding spindle and kinetochore associated protein 1 (*SKA1*) was identified as a target of anti-tumor *miR-10a-5p*. Overexpression of *SKA1* was detected in clinical specimens from patients treated with tyrosine kinase inhibitors and its expression contributed to cancer cell aggressiveness [22]. Interestingly, high expression of 4 genes targeted by anti-tumor miRNAs was significantly associated with poor prognosis of patients with RCC according to TCGA database analyses (*UHRF1*: *p* = 4.87E-06, *LOXL2*: *p* = 0.0343, *PLOD2*: *p* = 0.000855 and *SKA1*: *p* = 1.44E-07). These findings indicate that our miRNA-based approaches effectively identify molecular targets for RCC treatments.

Our recent studies showed that some miRNA passenger strands (*miR-144-5p*, *miR-145-3p*, *miR-149-3p*, *miR-150-3p* and *miR-199a/b-3p*) acted as anti-tumor miRNAs through their targeting of oncogenic genes in several cancers [10, 12–14, 16]. Our data represent an exception to the general concept of miRNA biogenesis and may offer new approaches to miRNA analysis. It has been reported by other research groups that passenger strands of miRNAs were functional in cancer cells. For examples, *miR-149-3p* inhibited cancer aggressiveness and metastasis in breast cancer [23]. Furthermore, *miR-21-3p* functions as a tumor suppressor via targeting methionine adenosyltransferase (*MAT*) in hepatocellular carcinoma [24]. In clear cell RCC, the *miR-514a-3p* expression level was significantly downregulated and behaves as a tumor suppressor through its targeting of epidermal growth factor receptor (*EGFR*) [25].

In RCC cells, we identified a total of 55 oncogenes that were putative targets of *miR-455-5p* and 33 that were likely targets of *miR-455-3p* (Tables 1 and 2). Among them, several genes have been reported to be involved in RCC pathogenesis. *KIAA0101* induced by erythropoietin, promoted cancer proliferation and migration, and higher expression of *KIAA0101* was associated with a poorer prognosis in RCC [26]. *VCAN* was overexpressed in clear cell RCC tissues and high expression of the gene was associated with metastasis and poorer survival after nephrectomy [27]. *PARVG* was also reported to be highly associated with RCC prognosis [28]. Furthermore, our research groups have revealed that *CENPF*, directly regulated by *miR-205-5p*, was overexpressed and involved in prostate cancer pathogenesis [29]. These target genes represent potential therapeutic targets for RCC. Our data suggested that both strands of *miR-455* duplex controlled several types of genes which contributed to cancer cell proliferation, invasion and migration. Thus, the elucidation of genes directly regulated by anti-tumor *miR-455* significantly help our understanding of pathogenesis in RCC.

Here, we focused on the SKA complex, which is a sub-complex of the outer kinetochore and attaches to spindle microtubules to maintain the metaphase plate during mitosis. The structure of the SKA complex is a coiled dimer formed by interaction between SKA1, SKA2 and SKA3 proteins [30]. Among them, C-terminal domains of SKA1 and SKA3 were especially important for microtubule binding and mitotic progression [30–32]. The SKA complex likely plays a pivotal role in the onset of anaphase in mitosis [33]. In cancer cells, including RCC, genes that regulate the cell cycle can mutate and produce excessive growth [34].

Our previous study showed overexpression of SKA1 in RCC clinical specimens, and its expression was associated with poor prognosis of RCC patients [22]. In this study, we demonstrated that expression of *SKA3* significantly contributed to RCC pathogenesis. Other studies showed that overexpression of *SKA1* and *SKA3* was related to cancer aggressiveness in several other cancers, including non-small cell lung cancer, prostate cancer, bladder cancer, gastric cancer, colorectal cancer and adenoid cystic carcinoma [35–40]. Previous study showed that overexpression of SKA2 was observed in breast cancer and lung cancer specimens and its expression was enhanced to cancer cell proliferation [41]. Interestingly, several oncogenic signaling pathways, including ERK1/2, AKT, FAK and SRC, are regulated by expression of *SKA1* in cancer cells [22]. These findings indicate that a member of the SKA complex might be a therapeutic target for RCC treatment.

In conclusions, both strands of the *miR-455* duplex, *miR-455-5p* and *miR-455-3p*, acted as anti-tumor miRNAs through their targeting of several oncogenes in RCC. Genome-wide gene expression analyses and *in silico* approaches revealed that *SKA1* and *SKA3* were regulated by these miRNAs. Overexpression of *SKA1* and *SKA3* was involved in RCC pathogenesis, and these molecules might be potential prognostic markers and therapeutic targets for RCC. Involvement of passenger strands of miRNAs in cancer pathogenesis is a novel concept that provides new approaches to the treatment of RCC pathogenesis.

## MATERIALS AND METHODS

### Clinical specimen collection, cell lines and cell culture

We collected tissues from 15 patients who were diagnosed with renal tumors and who underwent radical nephrectomy at Chiba University Hospital (Chiba, Japan) between 2012 and 2015. Table 4 shows clinicopathological features of the 15 patients. All patients were diagnosed with clear cell carcinoma. Prior to surgery, all patients agreed that their own specimens would be used for research and they signed informed consent documents. We used 2 human RCC cell lines (786-O and A498) obtained from the American Type Culture Collection (ATCC, Manassas, VA, USA) as previously described [29, 42, 43]. The 2 cell lines were cultured in RPMI1640 with 10% fetal bovine serum (FBS) (HyClone, Utah, USA).

**Table 4:** Characteristics of 15 patients with clear cell RCC

No.	Age	Gender	Grade	pT	INF	v	ly	e.g or ig	fc	im	rc	rp	s
1	65	F	G1>G2	T1a	a	0	0	e.g	1	0	0	0	0
2	59	M	G3>G2	T1b	a	0	0	e.g	1	0	0	0	0
3	70	M	G2>G3>G1	T1a	a	0	0	e.g	1	0	0	0	0
4	52	M	G2>G3	T1b	a	0	0	e.g	1	1	0	0	0
5	76	F	G2>G3	T3a	a	1	0	e.g	1	0	0	0	0
6	64	M	G2>G3>G1	T3a	b	1	0	ig	0	1	1	0	0
7	67	M	G2>G3>G1	T3a	b	1	0	ig	1	0	0	0	0
8	59	M	G3	T3a	b	1	0	ig	0	0	0	0	0
9	77	M	G1>G2	T1b	a	0	0	e.g	1	0	0	0	0
10	51	F	G2>G1>G3	T3a	b	1	0	ig	0	0	0	0	0
11	51	M	G2>G1	T1b	a	0	0	e.g	0	0	0	0	0
12	78	M	G2>G1>>G3	T1b	b	0	0	e.g	1	0	0	0	0
13	57	M	G2	T1b	a	0	0	e.g	0	0	0	0	0
14	54	M	G2>G1	T3a	a	0	0	e.g	0	0	1	0	0
15	74	F	G3	T3a	b	1	0	e.g	0	0	0	1	1

F, female; M, male; INF, infiltration; v, vein; ly, lymph node; e.g, expansive growth; ig, infiltrative growth; fc, capsular formation; im, intrarenal metastasis; rc, renal capsule invasion; rp, pelvis invasion; s, sinus invasion.

### Transfection of mature miRNA, siRNA or plasmid vectors into RCC cells

The following mature miRNA species were used in this study: mature miRNA and Pre-miR miRNA Precursors (*hsa-miR-455-5p*; P/N: PM10529 and *hsa-miR-455-3p*; P/N: PM11142; from Applied Biosystems, Foster City, CA, USA). The following siRNAs were used: Stealth Select RNAi siRNA, si-*SKA3* (HSS137458 and HSS176800; Invitrogen, Carlsbad, CA, USA), and negative control miRNA/siRNA (P/N: AM17111; Applied Biosystems). *SKA3* plasmid vectors were designed and created by Kazusa DNA Research Institution (Product ID: FHC28197; Kisarazu, Japan). miRNAs and siRNAs were incubated with Opti-MEM (Invitrogen) and Lipofectamine RNAiMax transfection reagents (Invitrogen), as previously described [29, 42, 43]. Plasmid vectors were incubated with Opti-MEM and Lipofectamine 3000 reagents (Invitrogen) by forward transfection with the manufacturer's protocol.

### Incorporation of *miR-455-5p* or *miR-455-3p* into the RISC

786-O cells were transfected with 10 nM miRNA by reverse transfection. After 48 h, immunoprecipitation was performed using a human Ago2 miRNA isolation kit (Wako, Osaka, Japan) with the manufacturer's protocol. Expression levels of *miR-455-5p* or *miR-455-3p* were determined by qRT-PCR. The expression data were normalized to the expression of *miR-26a* (product ID: 000404; Applied Biosystems), which was not influenced by either *miR-455-5p* or *miR-455-3p* transfection.

### Quantitative real-time reverse transcription polymerase chain reaction (qRT-PCR)

Total RNA was extracted with TRIzol reagent (Invitrogen) according to the manufacturer's protocol, as described previously [29, 42, 43]. The procedure for PCR quantification was described previously [29, 42, 43]. Expression levels of *miR-455-5p* (Assay ID: 001280) and *miR-455-3p* (Assay ID: 002244) were determined by TaqMan qRT-PCR (TaqMan MicroRNA Assay; Applied Biosystems) and normalized to *RNU48* expression (assay ID: 001006; Applied Biosystems). TaqMan probes and primers for *SKA1* (P/N: Hs00536843_m1; Applied Biosystems), *SKA3* (P/N: Hs00384927_m1; Applied Biosystems), *GAPDH* (an internal control; P/N: Hs02758991_m1; Applied Biosystems) and *GUSB* (an internal control; P/N: Hs00939627_ml; Applied Biosystems) were assay-on-demand gene expression products.

### Cell proliferation, migration and invasion assays

Cell proliferation assays were carried out with XTT protocols, migration was assessed by wound-healing assays and invasion assays were carried out with Matrigel-coated Boyden chambers, as previously described [29, 42, 43].

### Identification of putative target genes regulated by *miR-455-5p* and *455-3p* in RCC cells

To identify genes regulated by *miR-455-5p* and *miR-455-3p*, we used *in silico* analyses and genome-wide gene expression analysis, as described previously [29, 42, 43]. We used the TargetScanHuman 7.0 (August, 2015 release), TCGA, and OncoLnc databases to select and narrow down putative miRNA target genes [44–46]. An oligo microarray (Human Ge 60K; Agilent Technologies) was used for gene expression analysis. The microarray data were deposited into the GEO database (accession number: GSE106791).

### Western blot analysis

Cells were harvested 48 h after transfection, and lysates were prepared. Immunoblotting was performed with anti-SKA1 antibodies (1:500 dilution, SAB2701430; Sigma-Aldrich, St. Louis, MO, USA) and anti-SKA3 antibodies (1:1,000 dilution, ab175951; Abcam, Cambridge, UK). Anti-glyceraldehyde 3-phosphate dehydrogenase (GAPDH) antibodies (1:10,000 dilution, ab8245; Abcam) were used as an internal loading control. The procedures were described previously [29, 42, 43].

### Luciferase reporter assays

The partial wild-type sequence of the *SKA1* or *SKA3* 3′-UTR or that with deletion of the *miR-455-5p* and *miR-455-3p* target site was inserted between the *Sgf*I-*Pme*I restriction sites in the 3′-UTR of the *hRluc* gene in the psiCHECK-2 vector (C8021; Promega, Madison, WI, USA). The procedures were described previously [29, 42, 43].

### Immunohistochemistry

Tissue specimens were incubated overnight at 4°C with anti-SKA3 antibodies (1:50 dilution, ab175951; Abcam). The procedures were described previously [29, 42, 43].

### TCGA database analysis of RCC

To investigate the clinical significance of miRNAs and candidate target genes, we used TCGA cohort data based on RNA sequencing. Gene expression and clinical data were obtained from cBioportal and OncoLnc datasets (downloaded on November 15th, 2017).

### Statistical analysis methods

To analyze the relationships between two groups and the numerical values, we performed Mann-Whitney *U*-tests and paired *t*-tests. Spearman's rank test was performed to evaluate the correlation between the two groups. Relationships among more than three variables and numerical values were analyzed using Bonferroni-adjusted Mann-Whitney *U*-tests. Disease-free and overall survival were analyzed using the Kaplan–Meier method, and multivariable Cox hazard regression analyses with JMP software (version 13; SAS Institute Inc., Cary, NC, USA). Other analyses were performed using Expert StatView (version 5; SAS Institute Inc.).

## SUPPLEMENTARY MATERIALS AND FIGURES


