# Supplementary material for: Anti-tumor roles of both strands of the miR-455 duplex: their targets SKA1 and SKA3 are involved in the pathogenesis of renal cell carcinoma
Source: Oncotarget. 2018 Jun 1;9(42):26638–58. doi: 10.18632/oncotarget.25410 (PMC6003567; doi:10.18632/oncotarget.25410)
Supplement: Supplementary file 1 [file oncotarget-09-26638-s001.pdf]

Anti-tumor roles of both strands of the *miR-455* duplex: their targets *SKA1* and *SKA3* are involved in the pathogenesis of renal cell carcinoma

SUPPLEMENTARY MATERIALS

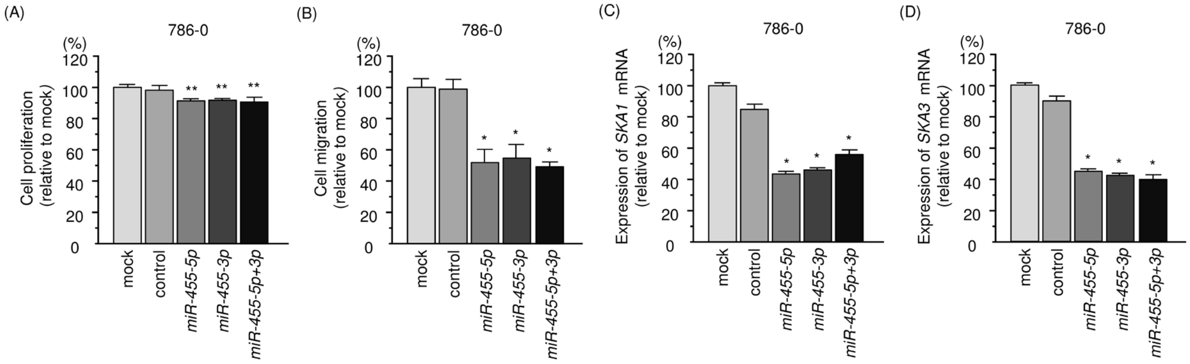

Supplementary Figure 1: The synergistic effects of *miR-455-5p* and *miR-455-3p* on RCC cells. **(A)** Cell proliferation was determined by XTT assay 72 h after transfection with 10 nM *miR-455-5p* and *miR-455-3p*. **(B)** Cell movement was assessed by migration assay 48 h after transfection with 10 nM *miR-455-5p* and *miR-455-3p*. **(C)** Expression levels of *SKA1* mRNA 48 h after transfection of 10 nM *miR-455-5p* and *miR-455-3p* into 786-O cells. **(D)** Expression levels of *SKA3* mRNA 48 h after transfection of 10 nM *miR-455-5p* and *miR-455-3p* into 786-O cells. $^{*}p < 0.0001$, $^{**}p < 0.01$.

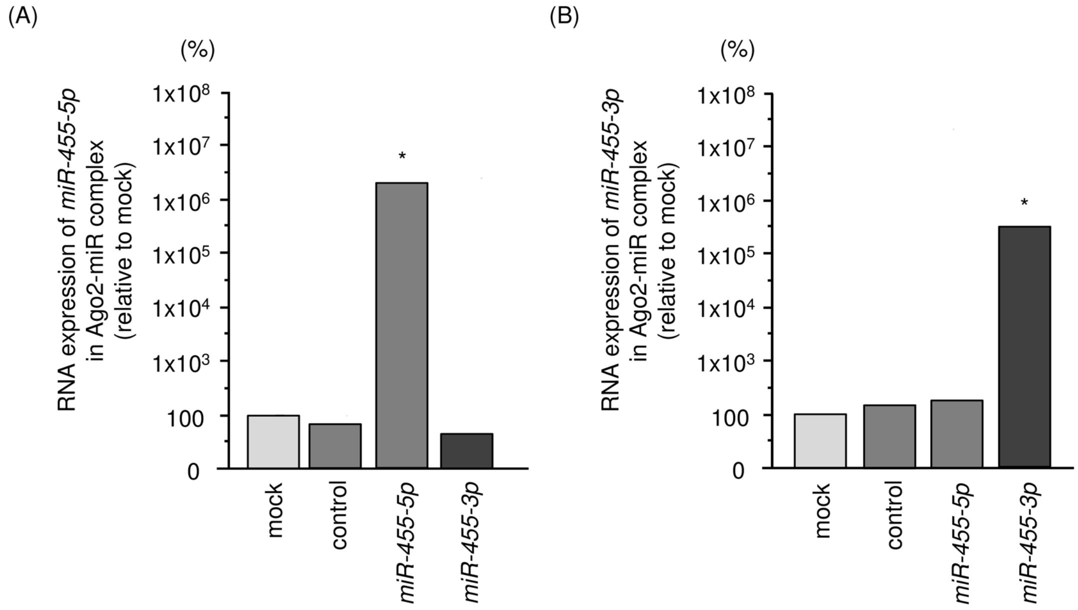

Supplementary Figure 2: Both strands of *miR-455-5p* and *miR-455-3p* were incorporated into the RISC. (A, B) Expression levels of *miR-455-5p* and *miR-455-3p* after transfection with *miR-455-5p* or *miR-455-3p* following immunoprecipitation by anti-Ago2 antibody. *, $p < 0.0001$.

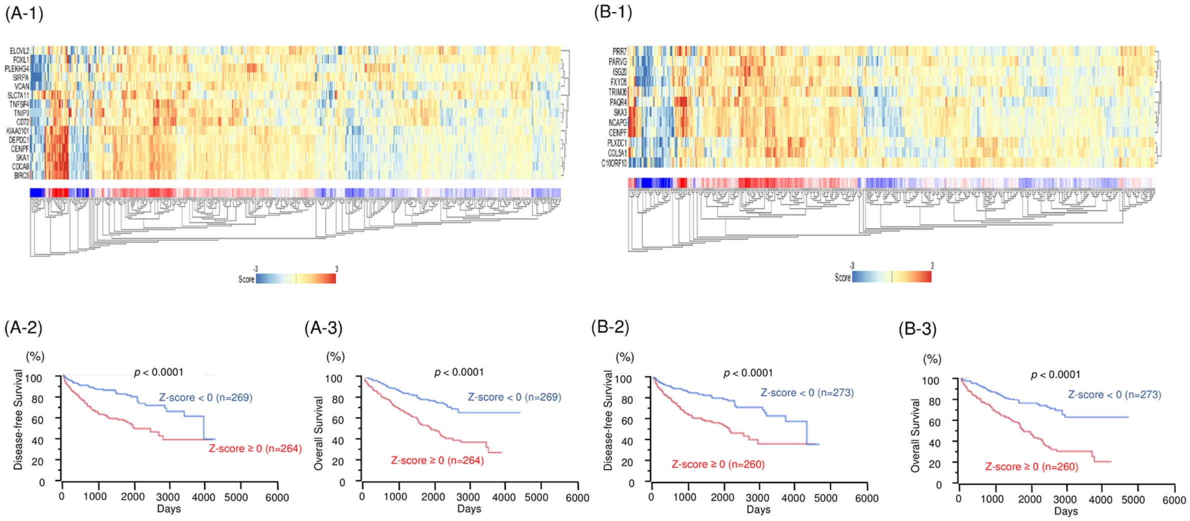

Supplementary Figure 3: Genomics Analysis and Visualization Platform were used for visualization of gene expression heatmaps (http://r2.amc.nl). The normalized mRNA expression values in the RNA sequencing data were processed and provided as Z-scores. **(A-1)** Gene expression heatmap of *miR-455-5p* targeted genes that had significant relationships with patient's prognosis (Table 2). (**A-2**, 3) Kaplan-Meier curves for DFS and OS in patients with low gene signature expression (Z-score < 0) and those with high gene signature expression (Z-score ≥ 0) of *miR-455-5p* targeted genes. **(B-1)** Gene expression heatmap of *miR-455-3p* targeted genes that had significant relationships with patient's prognosis. (**B-2**, 3) Kaplan-Meier curves for DFS and OS in patients with low gene signature expression (Z-score < 0) and those with high gene signature expression (Z-score ≥ 0) of *miR-455-3p* targeted genes.

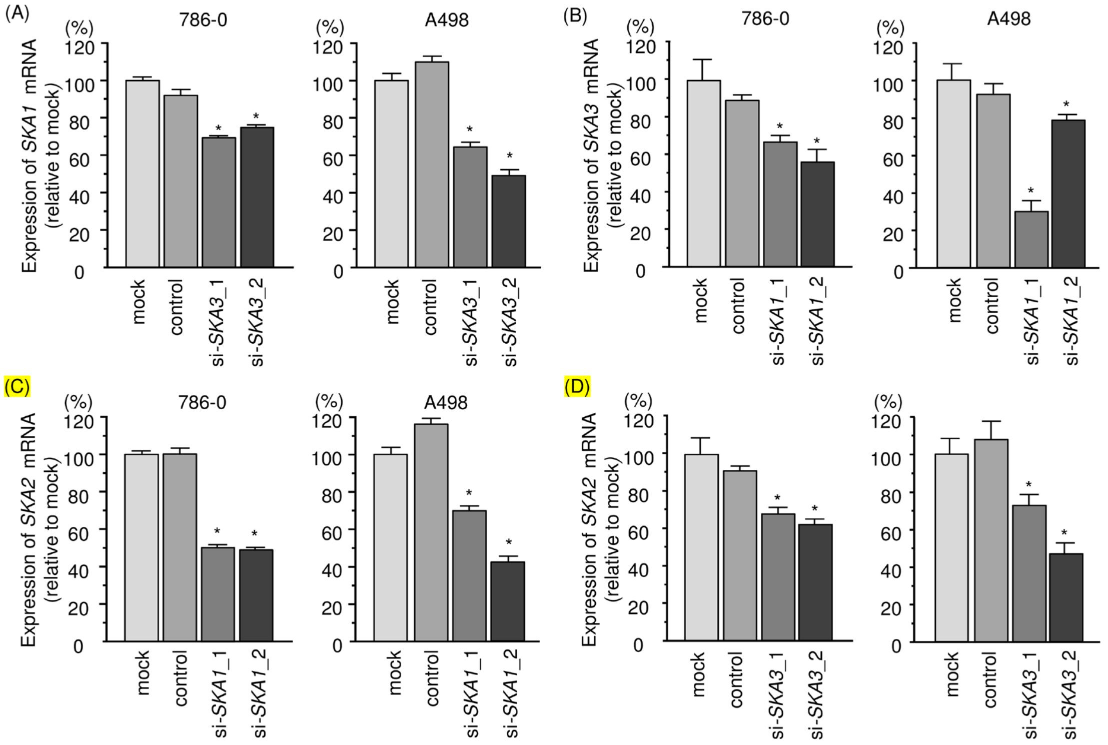

Supplementary Figure 4: Analysis of interaction of mRNA expression within *SKA* family. (A) *SKA1* mRNA expression 48 h after transfection with 10 nM si-*SKA3* into RCC cell lines. **(B)** *SKA3* mRNA expression 48 h after transfection with 10 nM si-*SKA1* into RCC cell lines. **(C)** *SKA2* mRNA expression 48 h after transfection with 10 nM si-*SKA1* into RCC cell lines. **(D)** *SKA2* mRNA expression 48 h after transfection with 10 nM si-*SKA3* into RCC cell lines. *GUSB* was used as an internal control. *, $p < 0.0001$.

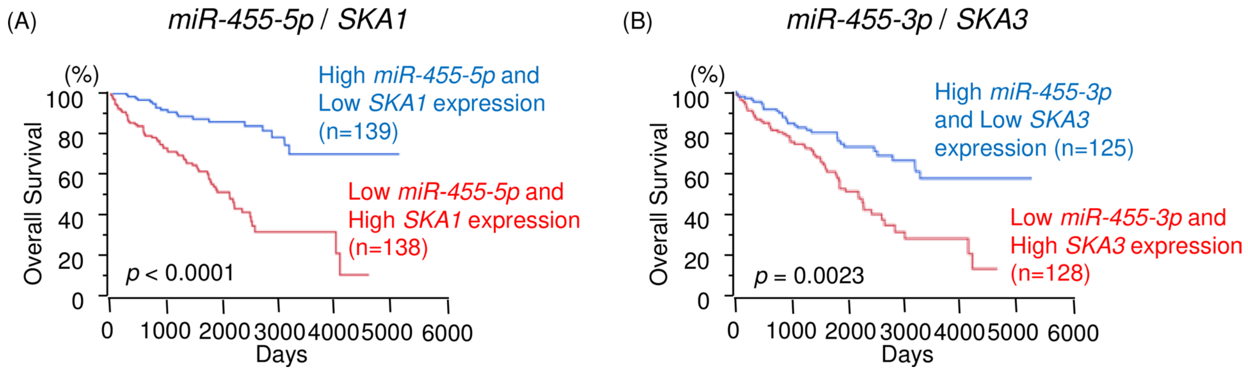

Supplementary Figure 5: Kaplan-Meier curves showed comparison of patients' prognosis when they were divided into two groups by combining the expression levels of *miRNA* and the regulated gene.
